# Association of NTCP polymorphisms with clinical outcome of hepatitis B infection in Thai individuals

**DOI:** 10.1186/s12881-019-0823-x

**Published:** 2019-05-22

**Authors:** Natthaya Chuaypen, Nongnaput Tuyapala, Nutcha Pinjaroen, Sunchai Payungporn, Pisit Tangkijvanich

**Affiliations:** 1 Center of Excellence in Hepatitis and Liver Cancer, Faculty of Medicine, Chulalongkorn University, Bangkok, 10330 Thailand; 2 Department of Radiology, Faculty of Medicine, Chulalongkorn University, Bangkok, 10330 Thailand; 3 Department of Biochemistry, Faculty of Medicine, Chulalongkorn University, Bangkok, 10330 Thailand

**Keywords:** Hepatitis B virus, Polymorphism, NTCP, Hepatocellular carcinoma, Susceptibility

## Abstract

**Background:**

Single nucleotide polymorphisms (SNPs) in the sodium taurocholate co-transporting polypeptide (NTCP) have been showed to be associated with natural history of hepatitis B virus (HBV) infection. However, it is unclear whether the SNPs are related to the clinical outcome of HBV infection in Thai individuals.

**Methods:**

The rs2296651 and rs4646287 polymorphisms of *NTCP* were determined by allelic discrimination using commercial *Taq*Man probes in blood samples of 1021 Thai individuals. These subjects included 610 patients with chronic HBV infection [CHB, 305 with hepatocellular carcinoma (HCC) and 305 without HCC], 206 subjects with spontaneous HBV clearance and 205 healthy controls who were age and gender-matched.

**Results:**

The frequencies of rs2296651 A minor allele in the CHB group, the HBV clearance group and healthy controls were 7.8, 7.3 and 13.9%, respectively. For rs4646287, the frequencies of T minor allele of the corresponding groups were 10.4, 8.0 and 9.5%, respectively. Compared with healthy controls, the frequencies of rs2296651 GA + AA genotypes were significantly lower in the CHB group (*P* &lt; 0.001) and in the HBV clearance group (*P* = 0.001). There was no difference in their distribution between the HBV clearance and CHB groups. Among the CHB group, the distribution of GA + AA genotypes in patients with HCC were significantly lower than in patients without HCC (*P* = 0.014). The frequencies of HBeAg positivity in patients harboring GG and GA + AA genotypes were 39.8 and 23.5%, respectively (*P* = 0.004). Among patients with HCC, the mean HBV DNA of the corresponding genotypes were 4.9 ± 1.3 vs. 2.7 ± 1.0 log_10_ IU/mL, respectively (*P* &lt; 0.001). There was no difference in genotype and allele frequencies of rs4646287 polymorphism among all studied groups.

**Conclusions:**

Our results showed that rs2296651 polymorphism was associated with a decreased risk of susceptibility to HBV infection and the development of HCC. These data suggest that the *NTCP* polymorphism might have an influence on natural history of HBV infection in Thai individuals.

This abstract was partly presented at the American Association for the Study of Liver Diseases (AASLD) Meeting 2018, November 9–13, 2018, in San Francisco, CA, USA and was published in Hepatology 2018; 68:1237A-1238A.

**Electronic supplementary material:**

The online version of this article (10.1186/s12881-019-0823-x) contains supplementary material, which is available to authorized users.

## Background

Hepatitis B virus (HBV) infection is a global public health problem with an estimated 240 million individuals are chronically infected with the virus [1]. In Thailand, the prevalence of chronic HBV infection is approximately 2.2% and the most common genotypes are genotypes C and B, respectively [2]. It has been generally recognized that the natural history of HBV infection is influenced by host and viral factors. After acute infection, some infected individuals are able to clear the virus, while a high proportion of patients have persistent infection leading to diverse clinical outcome including chronic hepatitis, cirrhosis and hepatocellular carcinoma (HCC) [1]. HBV is a partially double stranded DNA virus with highly complex genome organization and host specificity [3]. Recent data have demonstrated that HBV enters into the hepatocytes via a recently identified entry receptor, sodium taurocholate co-transporting polypeptide (NTCP), which specifically interacts with the pre-S1 region of HBV [4, 5]. This co-transporter, encoded by the *NTCP (SLC10A1*) gene with highly expressed on the sinusoidal membranes, plays a crucial role in bile duct enterohepatic circulation and regulating functions of the hepatocytes [6]. Previous data demonstrated that NTCP mutation (S267F) could result in a reduction of bile acid uptake and inhibition of HBV cell entry and viral replication [7].

Recently, single nucleotide polymorphisms (SNPs) in the *NTCP* gene have been found to be associated with the natural history and clinical outcome of HBV infection, although conflicting results were found. In particular, rs2296651 and rs4646287 of *NTCP* polymorphisms were associated with the risk of developing chronic HBV infection in Asian populations, while other studies reported no such association [8–15]. In addition, the association between these SNPs and HCC development is less clear with debatable results. These data emphasize the necessity to replicate such association studies in ethnically diverse populations. In this respect, the significance of these polymorphisms in Thai individuals remains to be elucidated. Thus, the aim of this study was to examine the effect and clinical relevance of rs2296651 and rs4646287 in HBV susceptibility and HCC development in Thai individuals.

## Methods

Thai patients who were diagnosed of chronic hepatitis B (CHB) at King Chulalongkorn Memorial Hospital, Bangkok, Thailand between January 2011 and December 2016 were recruited in this study. The diagnosis of CHB was defined by seropositivity for hepatitis B surface antigen (HBsAg) for at least 6 months and did not have any evidence of co-infection with hepatitis C virus and/or human immunodeficiency virus. Cirrhosis was diagnosed based on histologic examinations and/or imaging studies including ultrasound, CT scan and MRI. Patients with CHB were further divided into two groups on the basis of presence or absence of HCC. The diagnosis of HCC was established by typical imaging and/or histopathology according to the standard guideline [16]. Diagnostic criteria of HCC by dynamic CT or MRI were based on findings of focal liver lesions with hyperattenuation at the arterial phase and hypoattenuation at the portal phase. The baseline clinical characteristics of patients with HCC were recorded, which included sex, age, liver function tests, Child-Pugh classification, serum alpha-fetoprotein (AFP) level and the Barcelona Clinic Liver Cancer (BCLC) staging [17].

Subjects in two control groups included the spontaneous HBV clearance group and healthy controls, who were ethnically, age and gender-matched with patients with CHB. The spontaneous HBV clearance group was defined by HBsAg negativity but positivity for HBV core antibody (anti-HBc) and HBV surface antibody (anti-HBs) without receiving any antiviral therapy. Healthy controls were individuals without HBV serological markers (HBsAg and anti-HBc negativity) and were recruited from blood donors at National Blood Centre Thai Red Cross Society, Bangkok, Thailand.

The study was performed in accordance with the Declaration of Helsinki of human subjects. A written inform consent was obtained from each participant after the study was approved by the Institutional Review Board, Faculty of Medicine, Chulalongkorn University (IRB no. 046/60). Blood samples were collected at first visit of the participant and stored at –70 °C until further analysis.

HBV serological assays (HBsAg, HBeAg, anti-HBc and anti-HBs) were performed by commercially available enzyme-linked immunosorbent assays (Abbott Laboratories, Chicago, IL). Serum HBV DNA levels were tested by Abbott RealTime HBV assay (Abbott Laboratories, Chicago, IL) with the lower limit of detection of 10 IU/mL.

Phenol/chloroform extraction assay was applied to extract genomic DNA from 100 μl of buffy coat and extracted DNA was diluted in 30 μl of distilled water. *NTCP* rs2296651 (g.69778476 G &gt; A, GenBank accession number NC_000014.9) and rs4646287 (g.69796098 C &gt; T, GenBank accession number NC_000014.9) were determined by commercial *Taq*Man probes based on real-time PCR (Applied Biosystems, Bedford, MA) [8, 13]. Genotyping technique was performed by ABI fluorescence-based allelic discrimination assay as described previously [18].

The Chi-square and Fisher’s exact test were used to compare the distribution of categorical variables between groups, while the Mann–Whitney U-test, Student’s t-test or ANOVA were used to compare the continuous variables. Pearson’s Chi-square was used to test the deviation from Hardy-Weinberg equilibrium (HWE). Genotype and allele frequency models were used to assessed the associations of different genetic models. The comparison of odd ratio (OR) with 95% confidence interval (CI) between each group was measured by using MedCalc statistical software (Version 13.3.3; http://www.medcalc.org/calc/ odds_ratio.php). The log-rank test was calculated to compare the survival analysis by using the Kaplan–Meier method. The *P*-values less than 0.05 were indicated as statistical significance. For the genetic association analysis, the significance levels were further adjusted by Bonferroni correction to counteract the effect of multiple tests (α/n). In this study, the Bonferroni corrected *P* value was 0.025 (0.05/2). Statistical analysis was calculated using the SPSS software for Windows 22.0 (SPSS Inc., Chicago, IL).

## Results

Baseline clinical characteristics of 205 healthy controls, 206 individuals with spontaneous HBV clearance and 610 patients with CHB (including non-HCC and HCC) are summarized in Table 1. There was no difference in mean age and gender distribution among the studied groups. Among patients with CHB, 61 (20%) patients were diagnosed with cirrhosis and 229 (37.5%) patients had HBeAg positivity with average HBV DNA level of 4.6 log_10_ IU/mL.

**Table 1:** Baseline characteristics of subjects in the study

Characteristics	Healthy Controls (*n* = 205)	Spontaneous HBV clearance (*n* = 206)	Chronic HBV infection (*n* = 610)	*P*
Age (years)	51.5 ± 4.6	51.3 ± 5.9	52.6 ± 12.2	0.194
Sex				0.150
Male	128 (62.4)	137 (66.5)	425 (69.7)	
Female	73 (37.6)	69 (33.5)	185 (30.3)	
HBeAg				
Positive			229 (37.5)	
Negative			381 (62.5)	
HBV DNA (log_10_ IU/ml)			4.6 ± 1.9	

Data express as mean ± standard deviation or n (%), Differences between groups were tested by Chi-square test or

One-Way ANOVA as appropriate, *P &lt; 0.05

In this cohort, 25(8.2%) patients with non-HCC and 217 (71.1%) patients with HCC received oral antiviral treatment at the enrollment. Among patients with HCC, the initial tumor stages based on BCLC classification were as follows: 87 (28.5%), 106(34.8%) and 112(36.7%) patients in stages 0-A, B and C-D, respectively. The treatment modalities were as follows: 92 (30.2%) patients underwent surgical resection/liver transplantation, 189 (61.9%) patients received locoregional therapies and 24 (7.9%) patients received supportive care.

The genotype frequencies of rs2296651 and rs4646287 in the whole cohort were not deviated from Hardy-Weinberg Equilibrium (*P* = 0.770 and *P* = 0.411, respectively). Table 2 demonstrates allele and genotype frequencies the studied SNPs in each group of subjects. The CHB group had lower distribution of rs2296651 A allele compared with healthy controls [7.8% vs. 13.9%, odds ratio (OR) = 0.48, 95% confidence interval (CI): 0.34–0.69, *P* &lt; 0.001]. Similarly, the corresponding figures for GA genotype were 12.3 and 25.9%, respectively (OR = 0.40; 95% CI: 0.27–0.60; *P* &lt; 0.001). As the homozygote mutant AA genotype frequencies were rare in each studied group, we combined GA + AA genotypes for further analysis in a dominant model. Our result showed that the frequencies of GA + AA genotypes decreased significantly in the CHB groups compared with healthy controls (13.9% vs. 26.9%, OR = 0.44; 95% CI: 0.30–0.65; *P* &lt; 0.001).

**Table 2:** Genotype and allele frequencies of the studied SNPs in all groups

Polymorphisms	Healthy Controls (*n* = 205)	Spontaneous Clearance (*n* = 206)	Chronic HBV Infection (*n* = 610)	Chronic HBV infection vs. Healthy Controls		Chronic HBV infection vs. Spontaneous Clearance		Spontaneous Clearance vs. Healthy Controls	
				OR (95% CI)	*P*	OR (95% CI)	*P*	OR (95% CI)	*P*
rs2296651									
Genotype frequency									
GG	150 (73.1)	177 (85.9)	525 (86.1)	1	–	1	–	1	–
GA	53 (25.9)	28 (13.6)	75 (12.3)	0.40 (0.27–0.60)	&lt; 0.001* &lt; 0.001^a^	0.90 (0.57–1.44)	0.668	0.45 (0.27–0.74)	0.002* 0.001^a^
AA	2 (1.0)	1 (0.5)	10 (1.6)	1.43 (0.31–6.59)	0.648	3.37 (0.43–26.52)	0.248	0.42 (0.38–4.72)	0.485
GA + AA	55 (26.9)	29 (14.1)	85 (13.9)	0.44 (0.30–0.65)	&lt; 0.001* &lt; 0.001^a^	0.99 (0.63–1.56)	0.959	0.45 (0.27–0.74)	0.002* 0.001^a^
Allele frequency									
G	353 (86.1)	382 (92.7)	1125 (92.2)	1	–	1		1	–
A	57 (13.9)	30 (7.3)	95 (7.8)	0.48 (0.34–0.69)	&lt; 0.001* 0.001^a^	1.08 (0.70–1.65)	0.739	0.49 (0.31–0.77)	0.002* 0.002^a^
rs4646287									
Genotype frequency									
CC	168 (82.0)	175 (85.0)	495 (81.1)	1	–	1		1	–
CT	35 (17.0)	29 (14.0)	103 (16.9)	1.00 (0.66–1.52)	0.996	1.26 (0.80–1.96)	0.318	0.80 (0.47–1.36)	0.402
TT	2 (1.0)	2 (1.0)	12 (2.0)	2.04 (0.45–9.19)	0.355	2.12 (0.47–9.57)	0.328	0.96 (0.13–6.89)	0.968
CT + TT	37 (18.0)	31 (15.0)	115 (18.9)	1.05 (0.70–1.59)	0.798	1.31 (0.85–2.02)	0.219	0.80 (0.48–1.36)	0.414
Allele frequency									
C	371 (90.5)	379 (92.0)	1093 (89.6)	1	–	1		1	–
T	39 (9.5)	33 (8.0)	127 (10.4)	1.11 (0.76–1.61)	0.603	1.33 (0.89–1.99)	0.158	0.83 (0.51–1.35)	0.447

Data expressed as n (%), *OR* odds ratio, *CI* confidence interval, *Crude *P*-value, ^a^*P*-value (adjusted for age and sex)

Similarly, the frequencies of A allele, GA genotype and GA + AA genotypes in the HBV clearance group was significantly lower than those found in healthy controls (OR = 0.49; 95% CI: 0.31–0.77; *P* = 0.002, OR = 0.45; 95% CI: 0.27–0.74; *P* = 0.002, and OR = 0.45; 95% CI: 0.27–0.74; *P* = 0.002, respectively). However, their frequencies were comparable between the CHB and HBV clearance groups. Regarding rs4646287 polymorphisms, there was no difference in genotype and allele frequencies among all studied groups.

To determine whether the polymorphisms affected the clinical outcome of patients with CHB, we compared genotype and allele frequencies between patients with or without HCC (Table 3). The results showed that rs2296651 A allele was significantly lower in the HCC group compared to those without HCC (6.1% vs. 9.5%, OR = 0.61; 95% CI: 0.40–0.94; *P* = 0.026). Likewise, the frequencies of GA and GA + AA genotypes in the HCC group was significantly lower than that found in the non-HCC group (8.9% vs. 15.7%, OR = 0.52; 95% CI: 0.31–0.81; *P* = 0.010 and 10.5% vs. 17.3%, OR = 0.56; 95% CI: 0.35–0.89; *P* = 0.015, respectively. In subgroup analysis, the frequencies of GA and GA + AA genotypes were significantly lower in the HCC group compared with the cirrhosis group and non-cirrhosis group. However, the frequency of A allele was lower in the HCC group compared with the non-cirrhosis group but did not significantly differ from that of the cirrhosis group (Additional file 1: Table S1). In contrast, there was no difference in allele and genotype frequencies of rs4646287 between the HCC and non-HCC groups.

**Table 3:** Genotype and allele frequencies of the studied SNPs in patients with chronic HBV infection

Polymorphisms	The non-HCC group (*n* = 305)	The HCC group (*n* = 305)	HCC vs. non-HCC	
			OR (95% CI)	*P*
rs2296651				
Genotype frequency				
GG	252 (82.6)	273 (89.5)	1	–
GA	48 (15.7)	27 (8.9)	0.52 (0.31–0.86)	0.010* 0.009^a^
AA	5 (1.6)	5 (1.6)	0.92 (0.26–3.23)	0.900
GA + AA	53 (17.3)	32 (10.5)	0.56 (0.35–0.89)	0.015* 0.015^a^
Allele frequency				
G	552 (90.5)	573 (93.9)	1	–
A	58 (9.5)	37 (6.1)	0.61 (0.40–0.94)	0.026* 0.029^a^
rs4646287				
Genotype frequency				
CC	254 (83.3)	241 (79.0)	1	–
CT	42 (13.8)	61 (20.0)	1.53 (1.00–2.35)	0.053
TT	9 (3.0)	3 (1.0)	0.35 (0.09–1.31)	0.120
CT + TT	51 (16.8)	64 (21.0)	1.33 (0.88–1.99)	0.180
Allele frequency				
C	550 (90.2)	543 (89.0)	1	–
T	60 (9.8)	67 (11.0)	1.13 (0.78–1.63)	0.512

Data expressed as n (%), *OR* odds ratio, *CI* confidence interval, *Crude *P*-value, ^a^*P*-value (adjusted for age and sex)

**Fig. 1:**
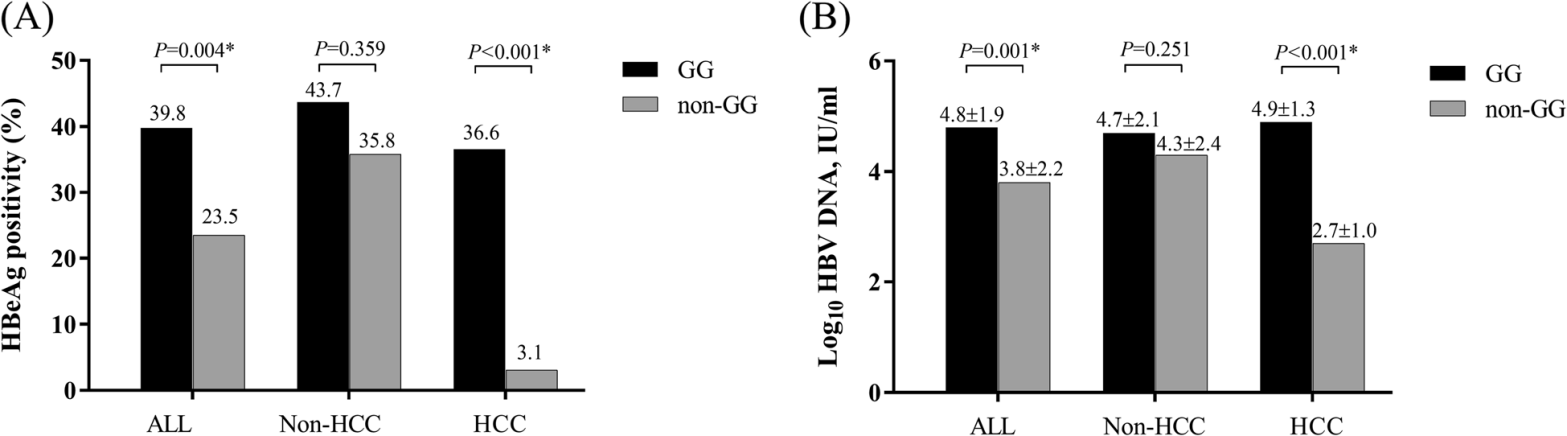
Viral markers in patients with CHB regarding rs2296651 genotypes (**a**) rates of HBeAg positivity (**b**) mean HBV DNA levels

To examine the correlation between the polymorphisms and viral replicative markers, HBeAg status and HBV DNA levels in patients with CHB regarding the presence of variants were compared. For rs2296651, the frequencies of HBeAg positivity in patients harboring GG and GA + AA genotypes were 209 (39.8%) and 20 (23.5%), respectively (*P* = 0.004). If separated patients with CHB into subgroups, HBeAg positivity rates of the corresponding genotypes in the non-HCC group were 110 (43.7%) vs. 19 (35.8%), respectively (*P* = 0.359) and in the HCC group were 99 (36.6%) vs. 1 (3.1%), respectively (*P* &lt; 0.001) (Fig. 1a). Among treatment-naïve cases, mean HBV DNA levels of all patients with GG and GA + AA genotypes were 4.8 ± 1.9 vs. 3.8 ± 2.2 log_10_ IU/mL, respectively (*P* = 0.001). The corresponding figures in the non-HCC group were 4.7 ± 2.1 vs. 4.3 ± 2.4 log_10_ IU/mL, respectively (*P* = 0.251), while the mean viral load in the HCC group were 4.9 ± 1.3 vs. 2.7 ± 1.0 log_10_ IU/mL, respectively (*P* &lt; 0.001) (Fig. 1b).

Regarding rs4646287, the frequencies of HBeAg positivity in patients harboring CC and CT + TT genotypes were 183 (37.0%) and 46 (40.0%), respectively (*P* = 0.593). In subgroup analysis, the frequencies of CC and CT + TT genotypes were comparable in the non-HCC group (42.1% vs.43.1%, *P* = 0.894) and in the HCC group (31.5% vs.37.5%, *P* = 0.372). There was no significant difference in mean HBV DNA levels regarding CC and CT + TT genotypes in all patients with CHB (4.5 ± 2.0 vs. 4.8 ± 1.9 log_10_ IU/mL, *P* = 0.207), in the non-HCC group (4.6 ± 2.1 vs. 4.8 ± 2.0 log_10_ IU/mL, *P* = 0.453) and in the HCC group (4.4 ± 1.4 vs. 4.9 ± 1.8 log_10_ IU/mL, *P* = 0.159).

As rs2296651 genotypes were associated with HCC development, its correlation with clinical parameters of patients with HCC was further evaluated (Additional file 2: Table S2). Patients with GA + AA genotypes had significantly higher frequency of female gender than patients harboring GG genotype. However, there were no significant differences between the two groups regarding other parameters. Moreover, the rs2296651 and rs4646287 genotypes were not associated with overall survival in patients with HCC (Additional file 3: Figure S1).

## Discussion

Accumulative evidence has suggested that host genetic variations play a crucial role in natural history and clinical outcome of patients with HBV infection. Recent data in Asian cohorts have shown that genetic variations in the *NTCP* genes might be associated with the risk of HBV infection and disease progression [8–13]. Thus, in this study we investigated the effect of NTCP polymorphisms on HBV susceptibility and HCC development in Thai population. Our report, recruited subjects with matched for age and genders among groups, showed that the frequencies of rs2296651 GA + AA genotypes were comparable between the CHB and HBV clearance groups, but significantly lower when compared with healthy controls. Specifically, individuals harboring these variants exhibited approximately 40–45% lower risk of HBV infection compared with healthy controls. These results could indicate that rs2296651 GA + AA genotypes were not linked to spontaneous HBV clearance. Instead, they were associated with increased resistance to HBV infection in Thai individuals. By contrast, any relationship between rs4646287 genotypes and HBV susceptibility or clearance was not found in this cohort. Although, previous data showed that NTCP expression was lower in liver tissues in individuals harboring non-CC genotypes that those with CC genotypes [13], such association was not established in a recent meta-analysis [10].

Our results regarding the protective role of this polymorphism are largely consistent with most previous reports conducted in Asian populations. For instance, Peng et al., Wang et al. and Wu et al. showed the low frequencies of rs2296651 GA + AA genotypes in Chinese Han patients with CHB compared with healthy controls [9, 10, 19]. Similarly, Hu et al. demonstrated that rs2296651 GA + AA genotypes were independently associated with decreased risk to CHB infection in Taiwanese individuals [8]. Another study by Nfor et al. also found that this genetic variation was linked to a decreased risk of HBV infection in Taiwanese women [20]. Likewise, Lee at al. recently showed that Korean individuals who harbored this variant were less susceptible to chronic HBV infection [15]. In contrast, Li et al. reported conflicting data that the variant was associated with susceptibility to HBV infection in Chinese Han patients compared with healthy controls [11]. The discrepancy from other reports including our cohort was not clear but might be related to relatively small number of samples included in the above-mentioned study. Moreover, Yang et al. did not find any association between the variant and risk of HBV infection, possibly due to lower frequency of the variant in their population of Eastern China [13]. Indeed, a possible protective role of rs2296651 polymorphism was further verified in a recent meta-analysis, demonstrating that this variant was inversely associated with the risk of HBV infection (OR = 0.593, *P* = 0.028) [10]. These data indicate that individuals harboring rs2296651 GA + AA genotypes have lower risk of CHB than those with GG genotype.

In this study, we found that the frequency of the A allele in healthy subjects was 13.9%, which was slightly greater than that found in most other Asian populations. Such high frequency of the minor allele might reflect the endemicity of HBV infection over the past decades before universal vaccination in Thailand. It should be mentioned that the minor allele frequency of rs2296651 varies among different ethnicities and geographic areas. For instance, previous data showed that its prevalence was 9.2% in Vietnamese, 8.7% in Taiwanese and 7.4% in Chinese, which were relatively higher than that found in Koreans (3.1%) [8, 21]. Interestingly, the frequency of the A allele among Han Chinese populations was relatively higher in Southern China (8–12.6%) in comparison to Central China (5.4–7.5%) and Northern China (2.4%) [10]. In contrast, the variant was reported to be very low or undetectable in regions where HBV infection is not endemic [22, 23]. Such information points out that the variant is particularly specific to Asian individuals, albeit varies among different ethnic populations, and might display its evolutionary advantage in conferring resistance to infection particularly in areas with high HBV prevalence.

In this study, we also investigated whether rs2296651 polymorphism might affect the development of HCC in Thai individuals. Our data showed that, among patients with CHB, GA + AA genotypes exhibited approximately 55% lesser distributed in the HCC group compared with the non-HCC. The same trend was also observed in subgroup analysis as these genotypes were less frequently found in the HCC group compared to non-HCC patients with or without cirrhosis. These results might suggest that individuals carried these genotypes had significantly lower risk of HCC compared with those with GG genotype, supporting their protective role in developing HCC among Thai patients. Previous data regarding the relationship between NTCP variants and HCC development in patients with CHB, however, has yielded conflicting results. Briefly, Hu et al. demonstrated the association of rs2296651 GA + AA genotypes persistently existed in Taiwanese patients with non-cirrhotic and cirrhotic HCC [8]. Likewise, Wang et al. and An et al. showed that Chinese Han individuals with the variant displayed lower risk of HCC compared with those carried the GG genotype [10, 14]. In contrast, Lee et al. showed that rs2296651 polymorphism did not correlate with a lower risk for HCC in Korean population [15]. Of noted, data supporting the protective role of this polymorphism for HCC development are cohorts conducted in Asian countries where the prevalent of the variant is relatively high such as in China, Taiwan and Thailand.

The mechanism regarding the effect of rs2296651 polymorphism on the risk of HCC remains unclear and needs further investigations. It has been shown that the rs2296551 variant leads to an amino acid change (S267F) in NTCP, resulting in functional alterations of the human NTCP receptor in transporting bile acids substrates and HBV susceptibility. Although its expression and membrane localization is not affected [22], the uptake of bile salt in the presence of this mutant is highly impaired as documented in previous clinical reports [24, 25]. Moreover, in vitro studies have demonstrated that NTCP function as a cellular receptor for HBV uptake is compromised by the variant [7, 22]. Thus, it is possible that the variant could lead to restriction of bile acid uptake and alter its homeostasis, resulting in reducing the possibility of cytotoxic bile salt accumulation in the hepatocytes [26]. As the likelihood of bile acid-mediated liver injury is attenuated, this alteration might lead to further reduction in fibrogenesis and finally diminish the risk of HCC development.

Interestingly, 10 patients with CHB (5 patients with HCC and 5 patients without HCC) in our cohort harbored AA genotype of rs2296651 polymorphism. This finding was in line with previous large-scale cohorts that a small proportion of HBV-infected individuals were shown to be homozygotes for this NTCP variant [8, 9]. As the variant is located at the critical region for HBV entry (amino acid 157–165 of NTCP), individuals who are homozygous carriers of this minor variant should be considered to have NTCP functional deficiency in supporting HBV infection [7]. Thus, the presence of patients with CHB carrying the homozygous variant may indicate existing minor alternative pathways for HBV entry or the possibility of pre-S1 mutants that enables HBV infection [8]. Alternatively, a recent in vitro study demonstrated that homozygous NTCP variant isolated from a patient exhibited a weak but authentic ability to facilitating HBV entry [27]. This novel finding supports the existence of HBV-infected individuals carrying the homozygous variant and may also explain our finding that declined HBeAg positivity and HBV viral load were particularly found in patients harboring GA + AA genotypes compared with those with GG genotype in patients with HCC.

This study had some limitations as being a retrospective cohort and investigating only 2 polymorphisms in the NTCP gene. In addition, occult HBV infection could not be completely excluded in individuals with spontaneous HBV clearance as serum HBV DNA levels were not performed. Finally, the serological testing for hepatitis delta (HDV) was not assessed in this cohort because of its low prevalence in Thailand.

## Conclusion

Our data demonstrated that rs2296651 polymorphism may be associated with the natural course of HBV infection in Thai population. Specifically, the GA + AA genotypes were associated with a decreased risk of susceptibility to HBV infection but were not associated with HBV clearance. In addition, the genotypes might be inversely associated with developing HCC in Thai patients with chronic HBV infection. Further studies are needed to confirm these findings and to verify the mechanisms by which the variant influences HCC development in individuals with diverse ethnic and clinical backgrounds.

## Additional files

Additional file 1: **Table S1.** Genotype and allele frequencies of the studied SNPs in patients with CHB (with and without cirrhosis) and HCC. (DOCX 17 kb)

Additional file 2: **Table S2.** Clinical characteristics of patients with HCC carried GG and GA + AA genotypes. (DOCX 15 kb)

Additional file 3: **Figure S1.** The effect of SNPs rs2296651 (A) and rs4646287 (B) on overall survival in patients with HCC. (TIF 769 kb)

## Abbreviations

CHB: Chronic hepatitis B
HBV: Hepatitis B virus
HCC: Hepatocellular carcinoma
NTCP: Sodium taurocholate co-transporting polypeptide
SNP: Single nucleotide polymorphisms
