# Supplementary material for: Association of NTCP polymorphisms with clinical outcome of hepatitis B infection in Thai individuals
Source: BMC Med Genet. 2019 May 22;20:87. doi: 10.1186/s12881-019-0823-x (PMC6532194; doi:10.1186/s12881-019-0823-x)
Supplement: Supplementary file 1 — Table S1. Genotype and allele frequencies of the studied SNPs in patients with CHB (with and without cirrhosis) and HCC. (DOCX 17 kb) [file 12881_2019_823_MOESM1_ESM.docx]

| **Additional file 1: Table S1** Genotype and allele frequencies of the studied SNPs in patients with CHB (with and without cirrhosis) and HCC | | | | | | | |
| --- | --- | --- | --- | --- | --- | --- | --- |
| **Polymorphisms** | **Non-cirrhosis** | **Cirrhosis** | **HCC** | **HCC vs. non-cirrhosis** | | **HCC vs. cirrhosis** | |
|  | **(n=244)** | **(n=61)** | **(n=305)** |  |  |  |  |
|  |  |  |  | **OR (95%CI)** | ***P*** | **OR (95%CI)** | ***P*** |
| **rs2296651** |  |  |  |  |  |  |  |
| Genotype frequency |  |  |  |  |  |  |  |
| GG | 203(83.2) | 49(80.3) | 273(89.5) | 1 | - | 1 | - |
| GA | 37(49.3) | 11(18.0) | 27(8.9) | 0.54 (0.32-0.92) | 0.023*  0.077^a^ | 0.44 (0.21-0.95) | 0.036*  0.068^a^ |
| AA | 4(1.6) | 1(1.6) | 5(1.6) | 0.93 (0.25-3.50) | 0.914 | 0.90 (0.10-7.85) | 0.922 |
| GA+AA | 41(16.8) | 12(19.7) | 32(10.5) | 0.58 (0.35-0.95) | 0.032*  0.149^a^ | 0.48 (0.23-0.99) | 0.048*  0.157^a^ |
| Allele frequency |  |  |  |  |  |  |  |
| G | 443(90.7) | 109(89.3) | 573(93.9) | 1 | - | 1 | - |
| A | 45(9.3) | 13(10.7) | 37(6.1) | 0.64 (0.40-0.99) | 0.049*  0.285^a^ | 0.54 (0.28-1.05) | 0.252 |
|  |  |  |  |  |  |  |  |
| **rs4646287** |  |  |  |  |  |  |  |
| Genotype frequency |  |  |  |  |  |  |  |
| CC | 203(83.2) | 51(83.6) | 241(79.0) | 1 | - | 1 | - |
| CT | 34(13.9) | 8(13.1) | 61(20.0) | 1.51 (0.95-2.39) | 0.078 | 1.61 (0.73-3.58) | 0.239 |
| TT | 7(2.9) | 2(3.3) | 3(1.0) | 0.36 (0.09-1.41) | 0.144 | 0.31 (0.05-1.95) | 0.215 |
| CT+TT | 48(19.7) | 12(19.7) | 64 (21.0) | 1.12 (0.74-1.71) | 0.586 | 1.13 (0.57-2.24) | 0.730 |
| Allele frequency |  |  |  |  |  |  |  |
| C | 440(90.2) | 110(90.2) | 543(89.0) | 1 | - | 1 | - |
| T | 48(9.8) | 12(9.8) | 67(11.0) | 1.13 (0.76-1.67) | 1.000 | 1.13 (0.59-2.16) | 1.000 |
| Data expressed as n (%), OR=odds ratio, CI=confidence interval, *Crude *P*-value, ^a^ *P*-value (adjusted for age and sex) | | | | | | | |
