# Supplementary material for: Association of NTCP polymorphisms with clinical outcome of hepatitis B infection in Thai individuals
Source: BMC Med Genet. 2019 May 22;20:87. doi: 10.1186/s12881-019-0823-x (PMC6532194; doi:10.1186/s12881-019-0823-x)
Supplement: Supplementary file 2 — Table S2. Clinical characteristics of patients with HCC carried GG and GA + AA genotypes. (DOCX 15 kb) [file 12881_2019_823_MOESM2_ESM.docx]

**Additional file 2: Table S2** Clinical characteristics of patients with HCC carried GG and GA+AA genotypes

|  | **rs2296651 genotypes** | | ***P* values** |
| --- | --- | --- | --- |
|  | **GG**  **N=273** | **GA + AA**  **N=32** |  |
| Age (years) | 58.2±10.1 | 58.2±13.7 | 0.980 |
| Sex (%)  Male  Female | 230(84.2%)  43(15.8%) | 22(68.8%)  10(31.3%) | 0.029* |
| TB (mg/dL) | 1.3±1.0 | 1.2±0.7 | 0.819 |
| Albumin (mg/dL) | 3.6±0.6 | 3.4±0.7 | 0.228 |
| AST (IU/L) | 83.3±78.7 | 82.4±66.5 | 0.950 |
| ALT (IU/L) | 57.8±59.2 | 51.6±38.7 | 0.568 |
| AFP (ng/mL) | 15169.8±11418.4 | 64563.4±42596.7 | 0.761 |
| Child-Puge score (%)  A  B  C | 183(67.6%)  78(28.6%)  12(4.4%) | 20(62.5%)  10(31.3%)  2(6.3%) | 0.829 |
| BCLC stage (%)  0-A  B  C-D | 78(28.6%)  94(34.4%)  101(37.0%) | 9(28.1%)  12(37.5%)  11(34.4%) | 0.936 |

ALT: Alanine transaminase, TB: Total bilirubin, AFP: Alpha-fetoprotein
