# Supplementary figures and images for: Association of NTCP polymorphisms with clinical outcome of hepatitis B infection in Thai individuals
Source: BMC Med Genet. 2019 May 22;20:87. doi: 10.1186/s12881-019-0823-x (PMC6532194; doi:10.1186/s12881-019-0823-x)

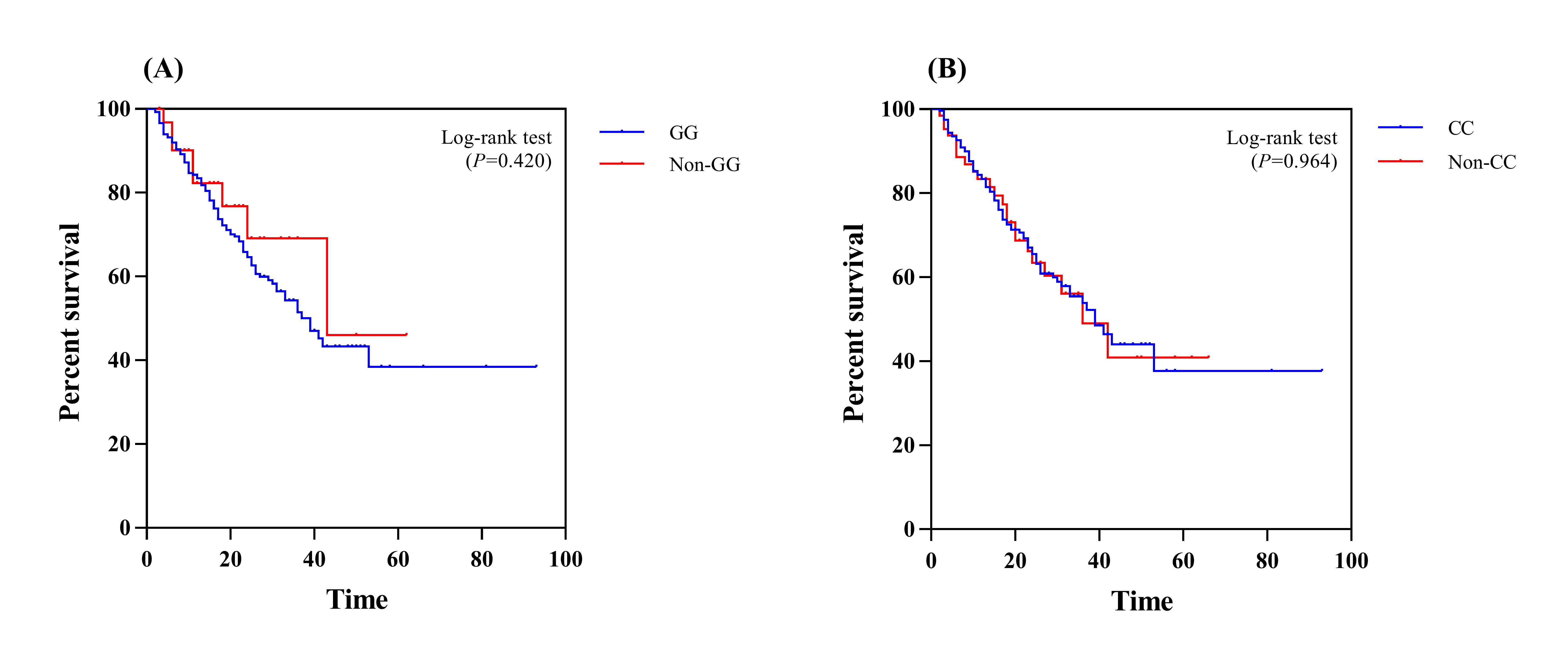

Supplement: Supplementary file 3 — Figure S1. The effect of SNPs rs2296651 (A) and rs4646287 (B) on overall survival in patients with HCC. (TIF 769 kb) [file 12881_2019_823_MOESM3_ESM.tif]
